# Clinical experience with Brexpiprazole in bipolar depression, off-label use. A six cases report

**DOI:** 10.1192/j.eurpsy.2025.2075

**Published:** 2025-08-26

**Authors:** S. Garcia Jorge, D. Fresno Fresno, P. Hervias Higueras

**Affiliations:** 1 Psychiatry, Hospital Universitario del Henares, Madrid, Spain

## Abstract

**Introduction:**

In 2015, the United States Food and Drug Administration (FDA) approved Brexpiprazole as an adjuvant treatment for adults with major depressive disorder and as a treatment for adults with schizophrenia. Although studies suggest that Brexpiprazole is an effective adjunctive treatment for major depressive disorder in Europe, the European Medicines Agency (EMA) approved Brexpiprazole only for the treatment of schizophrenia in adult patients.

**Objectives:**

To observe the safety, tolerability, and efficacy of brexpiprazole in patients with bipolar depression.

**Methods:**

We followed, during 2 months, six patients diagnosed with bipolar disorder who met DSM-5 criteria for a major depressive episode. Four of them were women, two men. All were being treated with mood stabilizing drugs (2 with valproic acid and 4 with lithium). The average age was 43 years.

Visits were conducted every 15 days. At each visit, we evaluated depressive symptom improvement, any adverse effects, and the emergence of manic or hypomanic symptoms.

All patients were informed of the off-label use of this drug and gave their consent.

**Results:**

Five out of six patients continued treatment throughout the study; only one patient discontinued due to adverse effects (amenorrhea). All patients who maintained treatment demonstrated a subjective improvement in depressive symptoms, as observed by both, clinicians and the patients themselves. No patients presented with manic or hypomanic symptoms suggestive of a shift to a manic pole.

**Conclusions:**

Although off-label, brexpiprazole may be beneficial for certain patients with depressive symptoms and a diagnosis of bipolar disorder. It displayed a good tolerability profile, with no observed shifts to mania in our small sample.

**Disclosure of Interest:**

None Declared

